# Peripapillary hyperreflective ovoid mass-like structure in arteritic versus nonarteritic anterior ischemic optic neuropathy

**DOI:** 10.3389/fopht.2026.1771903

**Published:** 2026-03-13

**Authors:** Marius B. Maartensson, Andreas Worm Bendtsen, Michael Stormly Hansen, Morten Jørgensen, Lea Lykkebirk, Steffen Hamann

**Affiliations:** 1 Department of Ophthalmology, Copenhagen University Hospital – Rigshospitalet, Copenhagen, Denmark; 2 Department of Clinical Medicine, University of Copenhagen, Copenhagen, Denmark; 3 Department of Neuro-Ophthalmology, Rothschild Foundation Hospital, Paris, France

**Keywords:** A-AION, anterior ischemic optic neuropathy, giant cell arteritis, NA-AION, OCT, peripapillary hyperreflective ovoid mass-like structure, PHOMS, PHOMS volume

## Abstract

**Introduction:**

A peripapillary hyperreflective ovoid mass-like structure (PHOMS) is an optical coherence tomography (OCT) specific finding associated with axoplasmic stasis in various optic neuropathies. Its occurrence in arteritic anterior ischemic optic neuropathy (A-AION) has not previously been described, and its prevalence and structural implications across AION subtypes remain incompletely understood.

**Materials & methods:**

In this exploratory retrospective, age-matched study, patients with A-AION and nonarteritic AION (NA-AION) enrolled in two prior prospective studies between March 2021 and August 2024 were included. All eyes had undergone high-resolution spectral-domain OCT imaging of the optic nerve head as well as visual acuity assessment on first visit of diagnosis. PHOMS was identified according to Optic Disc Drusen Studies (ODDS) Consortium criteria.

**Results:**

Twenty-two patients (22 eyes) were included, with 11 eyes in each AION subtype. PHOMS was observed in both A-AION and NA-AION. PHOMS was identified in 4 of 11 eyes (36.4%) with A-AION compared with 2 of 11 eyes (18.2%) with NA-AION. The estimated odds ratio for PHOMS presence in A-AION compared with NA-AION was 2.57 (95% CI, 0.36–18.33; p = 0.635). Bruch’s membrane opening diameter was similar between groups (A-AION: 1546.78 ± 134.14 μm; NA-AION: 1507.27 ± 133.95 μm), while mean peripapillary retinal nerve fiber layer thickness was larger in A-AION than in NA-AION. No differences were observed in macular ganglion cell layer volume, other retinal layer volumes, total retinal volume, or visual acuity between subtypes.

**Discussion:**

The observation of PHOMS across ischemic optic neuropathy subtypes suggests that PHOMS in AION may reflect axoplasmic disturbance related to the ischemic insult itself rather than the underlying disease etiology, although confirmation in larger, prospective studies is warranted.

## Introduction

Arteritic anterior ischemic optic neuropathy (A-AION) is a common and severe complication of giant cell arteritis (GCA). Rapid initiation of high-dose corticosteroid treatment is critical to prevent bilateral blindness and other systemic complications (1). Nonarteritic anterior ischemic optic neuropathy (NA-AION) is far more prevalent than its arteritic counterpart and may present with indistinguishable clinical features yet requiring an entirely different management strategy. Misclassification of these conditions can result in delayed or missed treatment of GCA or unnecessary exposure to high-dose corticosteroids in NA-AION. Consequently, there is a great need for biomarkers that can assist in the early differentiation between A-AION and NA-AION.

A peripapillary hyperreflective ovoid mass-like structure (PHOMS) was first defined in 2018 by Malmqvist et al. to describe a retinal optical coherence tomography (OCT) specific finding frequently observed in eyes with optic disc drusen (ODD) (2). Since then, PHOMS have been identified across a variety of ophthalmic and neurologic conditions, including papilledema, pseudopapilledema, myopia with optic disc tilt, and multiple sclerosis (3–5). A PHOMS is believed to represent herniation of swollen axons into the peripapillary space due to axoplasmic stasis (6, 7).

In NA-AION, a PHOMS is a well-documented phenomenon, with a reported prevalence of around 31-51% (8–10). It is particularly common in ODD-associated NA-AION (ODD-AION) (8, 9), and its prevalence appears to decline with age (10, 11). To date, however, a PHOMS has not been reported in the context of A-AION or GCA. This gap in knowledge is noteworthy, given that NA-AION is associated with crowding of the optic nerve head (ONH) and supposedly involves a local compartment syndrome (12), whereas A-AION results from complete or partial occlusion of the short posterior ciliary arteries due to arteritic thickening of the vessel walls, often causing sudden-onset ONH ischemia (13). These differing mechanisms could theoretically result in a higher degree of axoplasmic stasis - and thus more PHOMS – in NA-AION compared with A-AION.

Currently, it is unclear whether PHOMS are underreported or truly less prevalent in A-AION. In this hypothesis-generating study, we assessed the frequency and volume of PHOMS in small age-matched groups of with A-AION and NA-AION patients. The aim was to explore whether differences in PHOMS characteristics might help to distinguish between these conditions in the early diagnostic work-up. and, in such case, to generate preliminary data for the design of a larger, prospective study.

## Materials & methods

### Study population

This exploratory retrospective case-control study included aged-matched patients diagnosed with A-AION and NA-AION at a 1:1 ratio. A 1:1 matching ratio was elected to maximize statistical effect per included participant, which is particularly relevant in hypothesis-generating studies such as this. Study participants had previously been enrolled in two separate prospective studies: A-AION patients were enrolled from the GAME-study and NA-AION patients were enrolled from the NARROW-study, clinicaltrials.gov identifiers NCT05248906 and NCT05305079, respectively. All patients were recruited and examined at the Department of Ophthalmology at Rigshospitalet, Copenhagen, Denmark between March 2021 and August 2024. All procedures adhered to the Declaration of Helsinki and were approved by the Local Committee on Health Research Ethics of the Capital Region of Denmark (H-20032069 and H-20073063). Written informed consent was obtained from all participants prior to inclusion in the respective studies.

Inclusion in the current study required a clinical diagnosis of AION with availability of OCT imaging of the ONH and macula as well as relevant clinical data within 14 days of the clinical onset of AION. Clinical diagnosis of either A-AION or NA-AION was in all cases verified by an experienced neuro-ophthalmologist. Furthermore, in all A-AION cases, GCA diagnosis was clinically established by a rheumatologist and confirmed by at least one of the following examination modalities: temporal artery biopsy, temporal artery ultrasound, and 18F-fluorodeoxyglucose positron emission tomography/computed tomography (18F-FDG PET-CT).

Exclusion criteria comprised any history or evidence of prior optic neuropathy, including glaucoma, or significant retinal disease in the affected eye, and insufficient OCT image quality for reliable assessment of PHOMS.

### Data acquisition and image analysis

Spectral-domain OCT imaging protocols for identification of PHOMS varied slightly between patients, which either were 15x10° dense scans performed in enhanced depth-imaging (EDI) mode on Spectralis OCT; Heidelberg Engineering, Heidelberg, Germany or 5line cross OCT scans with 26x26 grid centered on optic disc on Triton; Topcon, Tokyo, Japan, **Table 1**. However, the assessment of structural OCT parameters were all from Spectralis OCT from Heidelberg such as 15x10° dense scans performed in enhanced depth-imaging (EDI) mode and 20° 24-line radial scans centered on the ONH were available, along with 30x25° scans centered on the fovea, summarized in **Table 2**. Data on best-corrected visual acuity (BCVA) at first visit were extracted from clinical records and converted to logarithm of the minimal angle of resolution (logMAR) using established formulas for both Early Treatment Diabetic Retinopathy Study (ETDRS) letter scores and Snellen fractions (14). For patients with NA-AION, automated perimetry (Octopus; Haag-Streit, Switzerland) was conducted and available at one-month visit after clinical diagnosis for all patients in this group.

**Table 1:** Demographic characteristics.

Variable	A-AION (Mean±SD)	NA-AION (Mean±SD)
Patient count	11	11
Eye count	11 / 22	11 / 22
PHOMS count	4 / 11	2 / 11
ODD count	0 / 10	2 / 11
OCT protocol ratio* (Heidelberg/Topcon)	10 / 1	10 / 1
Age (years)	75.73 ± 7.04	75.55 ± 6.44
Sex-ratio (M/F)	4 / 7	4 / 7

SD, standard deviation; A-AION, arteritic anterior ischemic optic neuropathy; NA-AION, nonarteritic anterior ischemic optic neuropathy; PHOMS, peripapillary hyperreflective ovoid mass-like structure; ODD, optic disc drusen; * (asterisk), OCT protocol for identification of PHOMS; M/F, Male/Female.

**Table 2:** A-AION vs NA-AION (per eye).

Measurement	n (A-AION)	A-AION (Mean±SD)	n (NA-AION)	NA-AION (Mean±SD)
PHOMS volume (mm³) [H]	3	0.186 ± 0.123	2	0.116 ± 0.148
pRNFL thickness (µm) [H]	10	188.40 ± 66.11	11	127.55 ± 44.02
mGCL volume (mm³) [H]	10	1.03 ± 0.13	10	0.92 ± 0.16
mIPL volume (mm³) [H]	10	0.88 ± 0.11	10	0.81 ± 0.11
mINL volume (mm³) [H]	10	0.97 ± 0.13	10	1.01 ± 0.07
mOPL volume (mm³) [H]	10	0.82 ± 0.06	10	0.83 ± 0.06
mONL volume (mm³) [H]	10	1.69 ± 0.18	10	1.80 ± 0.32
mRPE volume (mm³) [H]	10	0.37 ± 0.06	10	0.42 ± 0.09
Retinal volume (mm³) [H]	10	8.85 ± 0.57	10	8.75 ± 0.53
BMO diameter (µm) [H]	9	1546.78 ± 134.14	11	1507.27 ± 133.95
logMAR	11	0.17 ± 0.74	11	0.32 ± 0.41
Mean deviation (dB)			11	12.15 ± 7.68

SD, standard deviation; A-AION, arteritic anterior ischemic optic neuropathy; NA-AION, nonarteritic anterior ischemic optic neuropathy; PHOMS, peripapillary hyperreflective ovoid mass-like structure; mGCL, macular ganglion cell layer; mIPL; macular inner plexiform layer; mINL, macular inner nuclear layer; mOPL, macular outer plexiform layer; mONL, macular outer nuclear layer; mRPE, macular retinal pigment epithelium; pRNFL peripapillary retinal nerve fiber layer; BMO, Bruch's membrane opening; [H], OCT protocol from Heidelberg.

**Figure 1:**
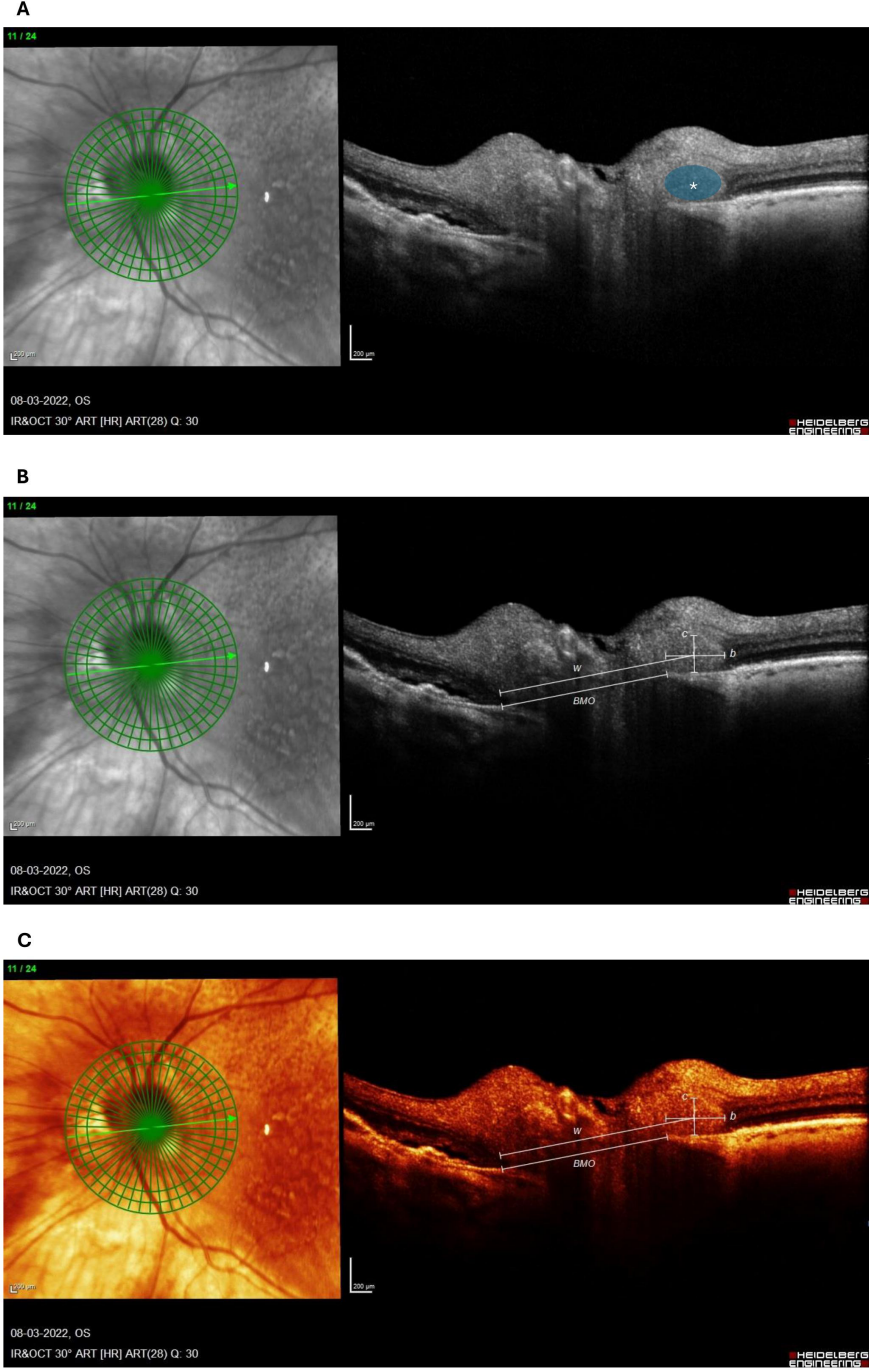
Dense optic nerve head OCT scan of an 86-year-old male with A-AION obtained 6 days after the ischemic insult. The PHOMS is marked by a blue circle with a white asterisk **(A)**. The method of PHOMS volume calculation $\;(2\pi^{2}abc$) is presented on a white on black OCT **(B)** and a heat contrast OCT **(C)** for better visualization. The following values were extracted: $b=\frac{537}{2}\;$; $c=\frac{213}{2}\;\;$; w=1578 and BMO=1357, where *b* = PHOMS fragment cross-sectional width divided by 2, *c* = PHOMS fragment cross-sectional height divided by 2, *w* = the distance between the far edge of BMO to the intersection of *b* and *c* and *BMO* is the Bruch’s membrane opening. Henceforth, the value of *a* is calculated through the following equation: $a=w-\frac{1}{2}\;BMO$. All values are in µm.

A PHOMS was identified according to the Optic Disc Drusen Studies (ODDS) Consortium criteria (2), defining it as a peripapillary hyperreflective mass-like structure located above Bruch’s membrane and associated with characteristic elevation or deflection of the overlying, adjacent retinal layers. OCT-scans were independently reviewed for the presence of PHOMS by two expert graders (M.J. and S.H.) blinded to the clinical diagnosis of A-AION or NA-AION, and any discrepancies were resolved through consensus discussion. Where available, 20° 24-line radial scans centered on the optic disc were used to measure PHOMS volume and Bruch’s membrane opening (BMO) diameters by experienced graders (A.W.B and M.B.M), which is visualized in **Figure 1**. This method is applied accordingly to previous descriptions by Jørgensen et al (11).

### Interpretation and statistical analyses

To create 1:1 age-matched pairs between the A-AION and NA-AION cohorts, we treated age matching as an optimal assignment problem. Pairs with an age gap >2 years were considered infeasible and assigned a prohibitively large cost, enforcing the ±2-year matching rule. We then applied the Hungarian algorithm to the resulting cost matrix to obtain the globally optimal one-to-one assignment i.e. the set of unique A-AION–NA-AION pairs that minimize the total summed age difference across the entire sample rather than relying on greedy or sequential matching.

This method yielded 11 unique, non-overlapping pairs, each meeting the ±2-year tolerance from the two prior studies. The complete set of paired observations is presented in **Supplementary Figure 1**, illustrating the alignment of the A-AION and NA-AION groups prior to subsequent comparative analyses.

The primary outcome was the comparative prevalence of PHOMS in A-AION versus NA-AION. For statistical analysis, a 2×2 contingency table was constructed, and Fisher’s exact test was applied, yielding odds ratios (OR) with 95% confidence intervals (95% CI).

Secondary measures included PHOMS volume, ODD prevalence, logMAR BCVA, peripapillary retinal nerve fiber layer (pRNFL) thickness, macular ganglion cell layer (mGCL) volume plus all retinal layers’ volume as well and BMO diameter. These measures were calculated and presented as mean ± standard deviation (SD) or as median (interquartile range), where appropriate.

All analyses were performed using R (version 2024.09.1 + 394).

## Results

### Study population and demographics

A total of 22 patients (11 with A-AION and 11 with NA-AION), age-matched pairwise within ± 2 years, were included in this study, **Supplementary Figure 1**. The matched dataset demonstrated a mean absolute age difference across all pairs to 1.27 years, with individual pairwise differences ranging from 0 to 2 years, indicating tight control of the primary matching variable. Notably, one pair was identically aged (Δ = 0), six pairs differed by only 1 year, and the remaining four pairs differed by 2 years, reflecting an even distribution of minimal age discrepancies.

Two patients – one in each group - exhibited bilateral AION but were limited to only right eyes for the final analysis, that included 11 eyes with A-AION and 11 eyes with NA-AION.

The two groups were very similar with an equal distribution of sexes (7 females and 4 males in each group) and age (75.73 ± 7.04 vs 75.55 ± 6.44 years), **Table 1**. With respect to identification of PHOMS, the two groups demonstrated equal availability of OCT protocols (Heidelberg/Topcon), as shown in **Table 1**. In addition, both groups were examined using the same OCT protocol (Heidelberg) for comparison for structural OCT parameters, where available, as detailed in **Table 2**.

### PHOMS prevalence in A-AION and NA-AION

PHOMS were identified in 4 of 11 eyes with A-AION compared with 2 of 11 eyes with NA-AION.

Notably, in the two patients with bilateral AION (one in each group), a PHOMS was detected in both the study eye and the fellow eye excluded from the analysis. ODD was identified in 2 of 11 eyes with NA-AION and in no eyes with A-AION, where dense EDI-OCT of the ONH was missing in a single eye as seen in **Table 1**. The two patients with ODD did not have PHOMS.

In this small sample, the estimated odds ratio of having a PHOMS between the A-AION and NA-AION groups was 2.57 (95% CI: 0.36–18.33, p = 0.635), but the result was not statistically significant, **Table 3**.

**Table 3:** Odds Ratio for PHOMS in A-AION vs NA-AION.

2×2 Contingency Table				
Group	PHOMS Present		PHOMS Absent	
A-AION	4		7	
NA-AION	2		9	
Summary: Odds Ratio for PHOMS (A-AION vs NA-AION)				
Group Comparison	Odds Ratio	Lower 95% CI	Upper 95% CI	p-value (Fisher)
A-AION vs NA-AION	2.57	0.36	18.33	0.635

PHOMS, peripapillary hyperreflective ovoid mass-like structure; A-AION, arteritic anterior ischemic optic neuropathy; NA-AION, nonarteritic anterior ischemic optic neuropathy; CI, Confidence interval; Fisher, Fisher’s exact test.

### Comparison of structural and functional characteristics in A-AION and NA-AION

PHOMS volumes were measured in the five patients with available radial optic disc scans. Within this small sample, volumes were similar across groups. Patients with A-AION (n=3) demonstrated a mean PHOMS volume of 0.186 mm^3^ (range 0.049 - 0.287 mm^3^), whereas patients with NA-AION (n=2) demonstrated a mean PHOMS volume of 0.116 mm^3^ (range 0.011 - 0.221 mm^3^).

BMO diameter was similar between groups with mean values and standard deviations of 1546.78 ± 134.14 in A-AION and 1507.27 ± 133.95 in NA-AION.

Mean peripapillary RNFL thickness was higher in A-AION (188.40 ± 66.11 µm) compared with NA-AION (127.55 ± 44.02 µm, p = 0.015). Macular GCL volume were similar between groups (A-AION: 1.03 ± 0.13 mm³, NA-AION 0.92 ± 0.16 mm³, p = 0.112), and no significant differences were found in volumes of other retinal layers or in total retinal volume.

Visual acuity measurements demonstrated similar mean logMAR values in A-AION (0.17 ± 0.74) and NA-AION (0.32 ± 0.41). Mean deviation on automated perimetry was available for only the NA-AION group, with an average of 12.15 ± 7.68 dB.

Structural and functional characteristics are summarized for each group in **Table 2**.

## Discussion

In this exploratory case-control study of PHOMS prevalence in AION, a PHOMS was observed more frequently in patients with A-AION due to GCA than in age-matched NA-AION patients. The difference, however, was not statistically significant, and the prevalence of PHOMS in A-AION patients in the current study matches the prevalence of PHOMS in NA-AION found in prior studies (8–10). This observation is hypothesis-generating and needs further exploration and power modelling for an ideal interpretation. However, it indicates that PHOMS formation is not isolated to the nonarteritic AION variant associated with a crowded optic disc anatomy and a likely component of compartment syndrome within the ONH in this study.

These results are consistent with the interpretation, that PHOMS in AION represents an epiphenomenon of axoplasmic stasis rather than a distinct pathological process with independent functional consequences (15). This aligns with current histopathologic and imaging-based models, which describe PHOMS as a manifestation of peripapillary axonal distension occurring in response to impaired axoplasmic flow (6, 7, 16). In this context, the appearance of PHOMS in A-AION likely reflects axoplasmic stasis secondary to ischemic compromise from posterior ciliary artery occlusion, rather than hypoperfusion leading to a local compartment syndrome, as proposed in NA-AION and often associated with a predisposed, crowded ONH (17) However, this cannot be demonstrated in the current study.

This study is the first to document PHOMS in A-AION using systematically collected OCT data, enabled by the availability of two well-defined and rigorously age-matched cohorts. The 1:1 age-matched design including the Hungarian algorithm is a notable strength, as it minimizes confounding of age on PHOMS prevalence.

At the same time, this study includes inherent limitations as retrospective studies induce selection bias, which was partly mitigated by the strict age matching of non-overlapping pairs. Furthermore, the modest sample size and slight differences in OCT scanning protocols between the original prospective datasets may introduce minor variability. Nonetheless, the consistent acquisition standards within each cohort and the uniform grading of PHOMS across all eyes support the robustness of the comparative analyses.

Larger studies incorporating a multi-modal approach and longitudinal follow-up may help clarify the pathogenesis and evolution of PHOMS across ischemic subtypes and whether its presence holds prognostic relevance beyond its role as a marker of axonal stasis.

However, this study generates a preliminary framework and warrants the need of elucidating the mechanisms underlying PHOMS formation across anterior ischemic optic neuropathies.

In summary, this study is the first to describe the presence of PHOMS in patients with A-AION, thereby extending current knowledge of PHOMS beyond the non-arteritic AION subtype. Although the sample size precludes definitive comparisons between AION subtypes, the observation of PHOMS in both arteritic and non-arteritic disease contributes to an improved understanding of PHOMS-formation in ischemic optic neuropathy.

## Data Availability

The raw data supporting the conclusions of this article will be made available by the authors, without undue reservation.

## Supplementary material

Supplementary Figure 1 1:1 Age-Matched pairs (A-AION vs. NA-AION). To ensure comparability between the two clinical subtypes, the cohorts were matched 1:1 by age using an optimal assignment procedure based on the Hungarian algorithm. This method yielded 11 unique, non-overlapping pairs, each meeting the ±2-year tolerance as visualized. The matched dataset demonstrated excellent balance: the mean absolute age difference across all pairs was 1.27 years, with individual pairwise differences ranging from 0 to 2 years, indicating tight control of the primary matching variable. Notably, one pair was identically aged (Δ = 0), six pairs differed by only 1 year, and the remaining four pairs differed by 2 years, reflecting an even distribution of minimal age discrepancies as described in the Materials & Methods section.
