# Supplementary figures and images for: Peripapillary hyperreflective ovoid mass-like structure in arteritic versus nonarteritic anterior ischemic optic neuropathy
Source: Front Ophthalmol (Lausanne). 2026 Mar 13;6:1771903. doi: 10.3389/fopht.2026.1771903 (PMC13021419; doi:10.3389/fopht.2026.1771903)

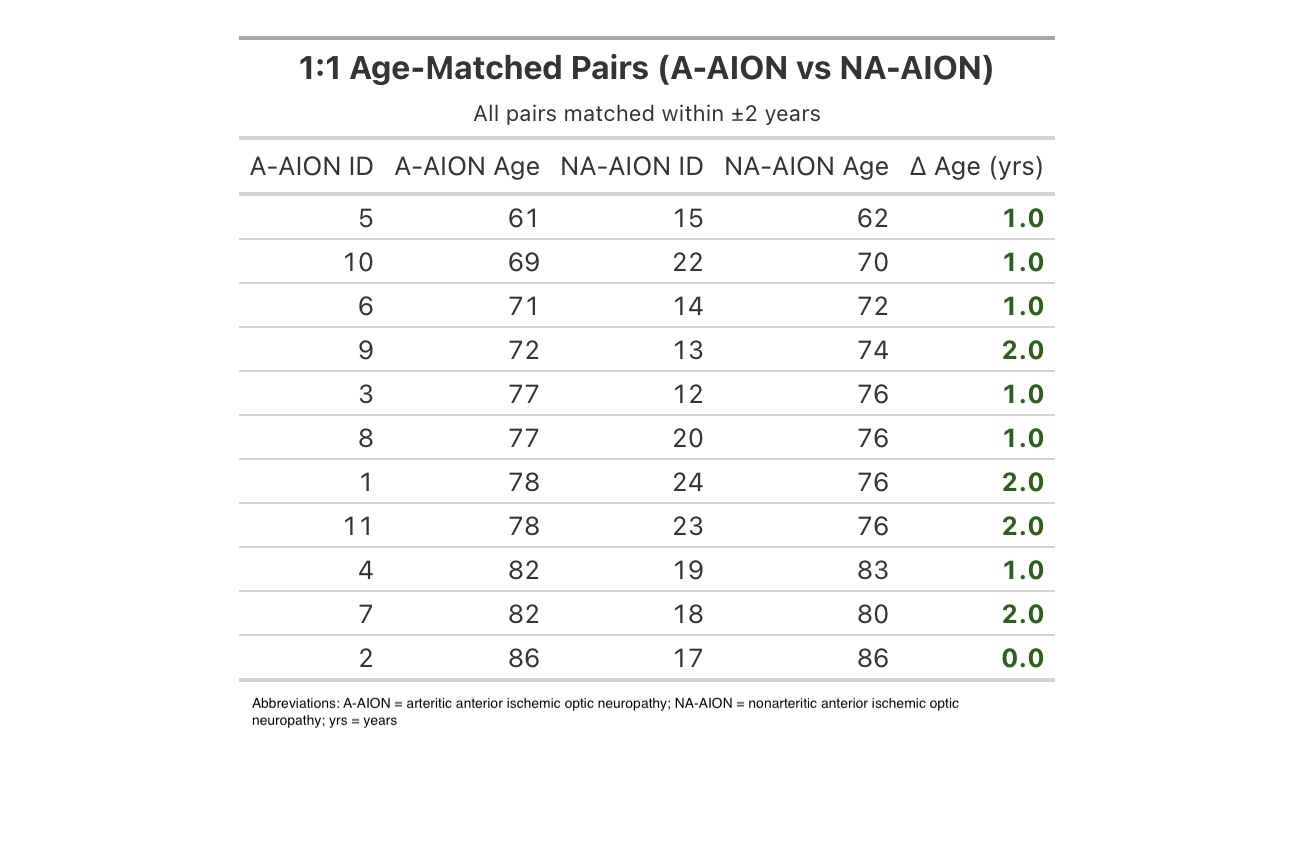

Supplement: Supplementary Figure 1 — 1:1 Age-Matched pairs (A-AION vs. NA-AION). To ensure comparability between the two clinical subtypes, the cohorts were matched 1:1 by age using an optimal assignment procedure based on the Hungarian algorithm. This method yielded 11 unique, non-overlapping pairs, each meeting the ±2-year tolerance as visualized. The matched dataset demonstrated excellent balance: the mean absolute age difference across all pairs was 1.27 years, with individual pairwise differences ranging from 0 to 2 years, indicating tight control of the primary matching variable. Notably, one pair was identically aged (Δ = 0), six pairs differed by only 1 year, and the remaining four pairs differed by 2 years, reflecting an even distribution of minimal age discrepancies as described in the Materials & Methods section. [file Image1.jpeg]
